# Decoding the decline: unveiling drivers of sarcopenia

**DOI:** 10.1172/JCI183302

**Published:** 2024-08-15

**Authors:** Allison M. Owen, Christopher S. Fry

**Affiliations:** 1 Center for Muscle Biology, University of Kentucky, Lexington, Kentucky, USA

## Abstract

There remains a critical need to define molecular pathways underlying sarcopenia to identify putative therapeutic targets. Research in the mechanisms of aging and sarcopenia relies heavily on preclinical rodent models. In this issue of the *JCI*, Kerr et al. implemented a clinically-relevant sarcopenia classification system of aged C57BL/6J mice, capturing sarcopenia prevalence across both sexes. The authors performed detailed physiological, molecular, and energetic analyses and demonstrated that mitochondrial biogenesis, oxidative capacity, and AMPK-autophagy signaling decreased as sarcopenia progressed in male mice. Sarcopenia was less prevalent in female mice with fewer alterations compared with the male-affected processes. The findings highlight factors beyond age as necessary for classifying the sarcopenic phenotype in rodent models, reveal sexual dimorphism across the trajectory of age-related declines in muscle mass and function in a commonly used rodent model, and provide insight into sex-dependent molecular alterations associated with sarcopenia progression.

## Establishing a mouse sarcopenia designation

Conserved across a majority of mammalian species, sarcopenia is the age-related loss of skeletal muscle mass and function, which is strongly predictive of mortality (1). Sarcopenic prevalence ranges between 10% and 40% of older adults dependent on the definition criteria; definitions including measures of muscle function in addition to muscle mass provide lower estimates of sarcopenia (2, 3). Despite ongoing updates to the clinical definition of sarcopenia (4), in preclinical rodent models, sarcopenia is usually designated by mouse or rat age or based only on muscle mass loss. During aging, the decline in muscle function exceeds deficits in muscle mass, underscoring the importance of functional assessment when defining sarcopenia. Given the importance of strength and function maintenance to mitigate age-related falls and injuries (5), several groups have successfully established preclinical frailty scoring criteria in C57BL/6 mice (6, 7). While compelling and discriminatory, these assessments are relatively burdensome in their assessment, requiring the longitudinal (or cross-sectional) assessment of a barrage of functional tests, including grip strength, inverted cling, treadmill, rotarod, and in vitro or in vivo muscle contractility. In this issue of the *JCI*, Kerr and colleagues provided a streamlined, clinically relevant definition of sarcopenia in aged (23–30 month old) C57BL/6J mice: at least two standard deviations below the mean of a young mouse reference group (4–9 month old) in measures of muscle mass, grip strength, and treadmill running time (8). Authors defined probable sarcopenia as exhibiting one deficit and sarcopenia as showing deficits in at least two out of the three criteria (8). Using their assessment, the authors determined the prevalence of sarcopenia to be approximately 20% in both 23–26 month old male mice and 27–28 month old female mice (8), comparable to the prevalence of sarcopenia is similarly aged humans (over 60 years old) (2, 3). Thus, this streamlined, clinically relevant protocol to classify sarcopenia phenotypes is feasible and reliable, enabling the improvement of sarcopenia-related animal studies beyond the common, age-alone designation, which is problematic given the approximately 20% prevalence observed in aged mice.

Preclinical rodent models provide a robust space to interrogate molecular mechanisms underlying sarcopenia. Taken together, prior literature has revealed multifactorial age-related alterations across pathways associated with loss of mitochondrial function, declines in anabolic factors (e.g., growth factors), elevated incidence of denervation, and increased inflammation in skeletal muscle (9–11). Abundant transcriptomic and proteomic changes have been identified across the lifespan, yet meaningful insight into sarcopenia has been limited with no consideration of muscle mass or function (9-11). The incorporation of a sarcopenic designation within the molecular profiling of aged muscle offers an important advancement to begin prioritizing putative therapeutic targets (8). As a further step, the inclusion of both male and female mice provided substantive advancement from prior work that was limited to male-only results (9-11), revealing a greater protection in female mice against declines in mitochondrial content and induction of protein catabolism (8).

## Sarcopenic-related impairment in mitochondrial function

**Figure 1:**
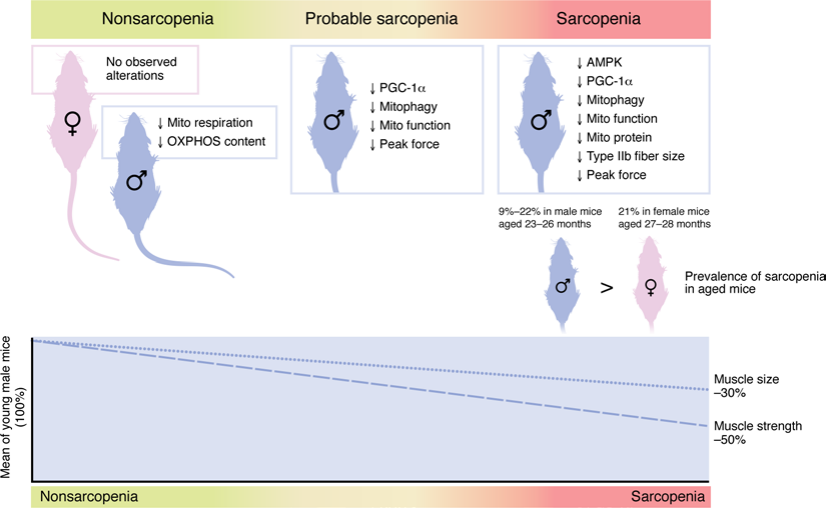
Sarcopenic deficits are associated with mitochondrial dysfunction in a sex-specific manner. Kerr et al. (8) showed that aged female mice displayed minimal molecular and cellular changes during the progression of sarcopenia, while male mice demonstrated several mitochondria-specific changes that may contribute to sarcopenic development. These changes included depressed oxidative capacity, AMPK-driven mitophagy, and PGC-1α–mediated mitochondrial biogenesis. Additionally, male mice displayed more prominent declines in muscle strength and physical performance as compared with muscle mass deficits during the progression of sarcopenia. Mito, mitochondrial.

Reduced muscle mitochondrial bioenergetic capacity is associated with declines in muscle contractility and physical function, as well as increased morbidity during aging (12–14). Similarly, Kerr and colleagues observed age-related declines in mitochondrial respiration in male mice (8). In an advancement, the authors found that reduced mitophagy as well as reduced mitochondrial biogenesis collectively contributed to low mitochondrial protein content. This reduction in mitochondrial content is likely, in part, responsible for deficits in oxidative capacity during the progression of sarcopenia (Figure 1). It should be noted that the observed association between mitochondrial capacity and muscle mass is most evident in male mice (8). Reductions in mitochondrial function may contribute to impaired skeletal muscle function not only in reduced energy production, but also in dysfunctional calcium regulation and production of reactive oxygen species, which results in oxidative damage (15). Similarly, in humans, mitochondrial energetics are negatively associated with age only in men (16). It should be noted that muscle mitochondrial energetics are lower in aged women compared with men (16), and mitochondrial deficits are strongly predictive of the elevated mobility impairment burden observed in older women (16).

Peroxisome proliferator-activated receptor γ coactivator 1-α (PGC-1α) promotes mitochondrial biogenesis and oxidative metabolism, and aging is associated with lower levels of PGC-1α (17, 18). Further, skeletal muscle–specific depletion of PGC-1α aggravates sarcopenia in aged mice (19), and its constitutive overexpression in skeletal muscle protects male mice from sarcopenia (20). Notably, Kerr and colleagues observed stepwise reductions in PGC-1α across nonsarcopenic, probable sarcopenic, and sarcopenic male mice (8). Consistent with lifelong muscle overexpression of PGC-1α, there appears to be important sex-specific consideration for the role of PGC-1α in sarcopenia (20).

## The contribution of canonical muscle atrogenes to sarcopenia

Atrogin1, also known as MAFbx, and MuRF1 are skeletal muscle–specific E3 ubiquitin ligases that regulate proteasomal degradation. Conflicting reports in human and rodent muscle on age-related expression changes in Atrogin1 and MuRF1 limit their conclusive designation as molecular effectors of sarcopenia (21). Genetic knockout of MuRF1 preserves muscle mass but not muscle function during aging (22), and its overexpression directly promotes muscle atrophy in young mice (23). Kerr and colleagues did not observe increases in MuRF1 or Atrogin1 in aging or in the progression of sarcopenia in the current study (8), challenging further the direct contributions of these canonical atrogenes in the etiology of sarcopenia. Strikingly, MuRF1 exhibited lower expression in sarcopenic male mice (8), similar to findings in aged rats (24). In the current report, female mice were protected against changes in Atrogin1 and MuRF1 during aging and during the development of sarcopenia (8). The loss of muscle proteostasis remains a hallmark of aging, yet the current results suggest that Atrogin1 and MuRF1 are unlikely to be critical determinants during sarcopenic progression. Similar to mitochondrial energetics, the findings from Kerr et al. (8) further underscore the necessity of sex-specific considerations during interrogation of molecular mechanisms underlying the development of sarcopenia.

## Concluding remarks

This report by Kerr et al. (8) highlights the importance of defining sarcopenia phenotypes based on muscle functional deficits rather than solely on age. The authors introduce a practical approach to classify mice into nonsarcopenic, probable sarcopenic, and sarcopenic categories using accessible methods such as treadmill running and grip strength assessment, which can be applied in future research studies. Using this model in male and female mice, the authors demonstrated a higher tendency of sarcopenia in males than females, associated with more rapid declines in muscle strength than in muscle size, which aligns with findings in human studies (2,3). The study provides evidence that alterations in bioenergetics (deficits in PGC-1α–mediated mitochondrial biogenesis and oxidative capacity) and AMPK-driven autophagy, are likely contributors to sarcopenia, with these mechanisms operating in a sex-dependent manner. Interestingly, the study did not find involvement of atrogin 1–associated proteasome ubiquitination signaling in sarcopenia. Broadly, this work underscores the importance of validating phenotypes based on functional criteria and considering sex as a critical variable in the design of rodent models of disease, aiming to enhance their translational relevance.

## Version 1 08/15/2024

Electronic publication
